# Serum Analysis in Patients with Temporomandibular Disorders: A Controlled Cross-Sectional Study in Norway

**DOI:** 10.1155/2019/1360725

**Published:** 2019-10-07

**Authors:** Kordian Staniszewski, Henning Lygre, Trond Berge, Annika Rosén

**Affiliations:** ^1^Department of Clinical Dentistry, University of Bergen, Bergen, Norway; ^2^Department of Oral and Maxillofacial Surgery, Haukeland University Hospital, Bergen, Norway

## Abstract

Temporomandibular disorder (TMD) is characterized by pain and dysfunction in the temporomandibular join (TMJ) and the masticatory apparatus. Associations with autoimmune diseases, inflammatory conditions, and nutrition deficiencies have been reported in previous studies of TMD patients. To evaluate essential proteins, hormones, electrolytes, and vitamins in serum from TMD patients, a standard blood sample analysis was performed in 60 TMD patients and 60 healthy controls matched for age and gender, retrieving 19 different analyses. We found that TMD patients had significantly higher values of hemoglobin (*p*=0.036), cobalamin (*p*=0.023), albumin (*p*=0.005), parathyroid hormone (PTH) (*p*=0.038), and vitamin D (*p*=0.005), and significantly lower values of creatinine (*p*=0.006) and potassium (*p*=0.011), compared to controls. In the TMD group, most of the determinants had a wider range, and several subjects, compared to the control group, had values outside the normal reference area. However, most of the TMD patients and controls had values within normal biological range. Our findings could not associate any severe systemic disease, malnutrition, or systemic inflammation with the TMD. Results from our study suggest that serum analyses should neither be used as a biomarker of TMD nor a diagnostic tool for an individual subject with TMD.

## 1. Introduction

Temporomandibular disorder (TMD) is characterized by pain and dysfunction in the masticatory apparatus and the temporomandibular joint (TMJ). A significant higher prevalence of comorbidities, such as degenerative arthritis, gastrointestinal symptoms, fibromyalgia, depression, and fatigue, has been revealed in this group of patients [1]. Autoimmune diseases and inflammatory conditions have been associated with TMJ disease (TMJD) in a recent hospital-based case-control study [2]. Summary from the Prospective Evaluation and Risk Assessment (OPPERA) project describes the etiology of TMD as complex and multifactorial, where pain sensitivity, biopsychosocial effects, and comorbidity are some of the contributing factors [3]. The prevalence of TMD is higher in women, who had a two-time higher risk in development of TMD compared to men [4]. In addition, pain intensity has been reported to be greater in women compared to men [5].

In TMD patients, few studies analyzing health status in serum have been published previously. Significantly, higher levels of parathyroid hormone (PTH) have been observed in TMD patients compared to a control group [6]. Patients with TMD as well as TMJD showed high prevalence of nutrition deficiencies, including iron, ferritin, vitamin D, vitamin C, vitamin B1, vitamin B6, vitamin B12, and folate [7, 8]. High levels of C-reactive protein (CRP) have been associated with inflammation in the TMJ in patients with rheumatoid arthritis (RA) [9]. On the other hand, no difference in levels of CRP was reported in patients with persistent TMJ pain, compared to controls [10]. Low levels of vitamin D have been associated with incidence of chronic pain [11] and chronic pain-related comorbidities, including sleep deprivation and depression [12]. One study revealed a high prevalence of vitamin D deficiencies in TMD patients; however, there were no significant differences in vitamin D or calcium levels in those patients compared to a control group [6].

Levels of the current hormones, electrolytes, and vitamins can easily be detected by blood sampling. Standardized serum tests can be used in diagnostics and evaluation of patient's health to detect diseases [13]. Laboratory serum analyses are also useful in revealing inflammation and autoimmune diseases and may indicate severity of the disease [14].

To our knowledge, the present study is the first to reveal as many as 19 different determinants in serum from TMD patients at the same time. The primary aim of the present study was to evaluate essential proteins, hormones, electrolytes, and vitamins in serum from TMD patients. Our hypothesis is that TMD patients have significantly different values of essential proteins, hormones, electrolytes, and vitamins in serum, compared to a healthy control group, and this may influence TMD symptoms.

## 2. Materials and Methods

### 2.1. Study Design

The present study was a controlled cross-sectional study, as part of a multidisciplinary investigation of TMD patients at Haukeland University Hospital (HUS) in Bergen, Norway [15]. Six different specialists including two dental specialists, one anesthesiologist, one psychologist, one physiotherapist, and one radiologist examined the patients. Pain-related symptoms and dysfunction (both general and TMD-related), general health status, psychosocial factors, previous treatment and medication, and the duration of pain and disease were disclosed.

Ethical approval was granted by the Regional Ethical Review Board South East (2015/930), in accordance with the Helsinki Declaration (1964). All subjects submitted written informed consent as a prerequisite for participating in the study.

### 2.2. Participants

The study population consisted of 60 TMD patients with severe symptoms and long-term pain and 60 healthy controls matched for age and gender. A previous study regarding stress and HPA axis regulation, involving the majority of the present study group, has been published [16]. The TMD patients were referred by their general practitioner (GP) to the project from all health regions in Norway, during the years 2013–15, and were clinically examined and evaluated consecutively. Patients were included, examined, and evaluated based on the severity and duration of symptoms, both for pain and dysfunction and for consequences. The six specialists at HUS, representing several disciplines, performed the examination and created an individual treatment proposal for each patient. The investigation included pain intensity and duration, functional impairment (general and jaw-specific), effect on quality of life, and presence of extended periods of sick leave. The inclusion criterion was long-term TMD-related pain. Furthermore, inclusion was based on the examination; thus, patients with and without functional impairment were included. The exclusion criteria were non-TMD-related orofacial pain, relevant drug dependence problems, and obvious psychiatric diagnoses. A healthy age- and gender-matched control group was recruited and examined during 2016.

The control group consisted of employees and students from the Department of Clinical Dentistry at the University of Bergen and was not a part of the study research group; additionally, there were a few subjects from the general population in Bergen. The inclusion criterion was age- and gender-matched with the patient group. The exclusion criteria were TMD symptoms, musculoskeletal pain, and symptoms in the head and neck area.

### 2.3. Serum Analyses

A standard blood sample analysis was conducted at Haukeland University Hospital and analyzed at the Laboratory for Clinical Biochemistry. The serum analyses retrieved 19 different analyses consisting of essential proteins, hormones, electrolytes, and vitamins. Those were hemoglobin (Hb), erythrocyte volume fraction (EVF), mean corpuscular volume (MCV), homocysteine, transferrin receptor (TfR), thyroid stimulating hormone (TSH), free thyroxine (FT4), parathyroid hormone (PTH), cobalamin, folate, C-reactive protein (CRP), creatinine, estimated glomerular filtration rate (GFR), sodium, potassium, calcium, gamma-glutamyl transferase (GT), albumin, and 25 (OH) vitamin D3 (vitamin D). CRP levels lower than 1 mg/L were registered as 1 mg/L due to limitations in the laboratory.

### 2.4. Statistical Analyses

Statistical analyses were performed in STATA. Mean, range, and standard deviation (SD) were calculated for all variables in both groups. Since our study matched age and gender between the groups, paired *t*-tests were appropriate to calculate *p*-value of no difference in all determinants from the serum analyses between the TMD group compared to the control group and between subgroups of women and men in the TMD group compared to women and men control group. Linear correlations (*R*) with associated *p*-values between CRP and pain parameters and between vitamin D and pain parameters, as well as between vitamin D and PTH, were calculated in both groups.

## 3. Results

### 3.1. Demographic Data

**Figure 1:**
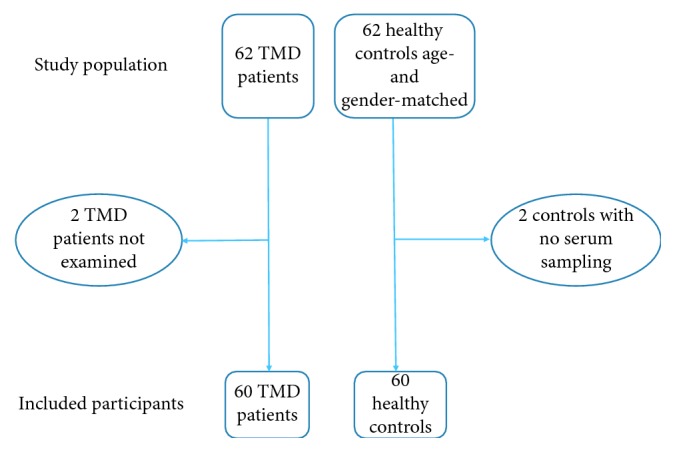
Flow chart of the study population: TMD patients and healthy controls.

The group of 60 TMD patients consisted of 51 women and 9 men, all affected with severe TMD symptoms. Mean pain duration in the patient group was 11 years (ranged 1–40 years). Mean age of the patient group was 45 years (ranged 20–69 years). Registered diagnoses on our clinical examination of the TMD group were fibromyalgia (*n* = 8), migraine (*n* = 12), and chronic fatigue (*n* = 4). The control group was matched for age and gender and had a mean age of 46 years (ranged 23–71 years). The study population is presented in Figure 1.

### 3.2. Serum Analyses

**Figure 2:**
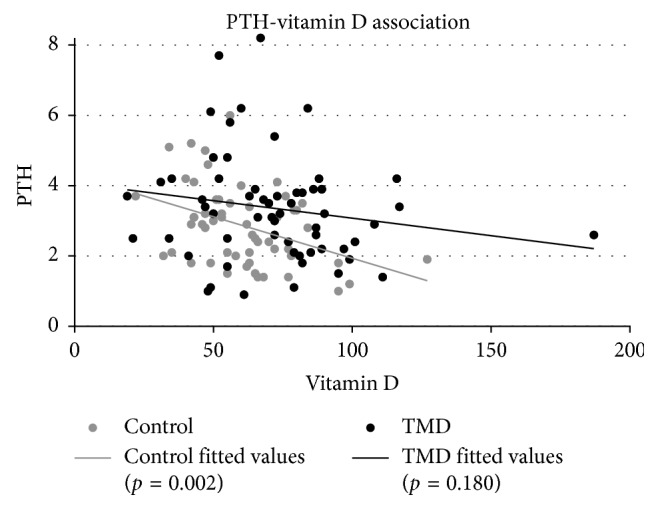
Linear correlation between parathyroid hormone and vitamin D in the TMD group (black) and the control group (grey). Low levels of vitamin D were significantly correlated (*p*=0.002) with high levels of PTH in the control group; however, this correlation was nonsignificant in the patient group.

Results revealed that TMD patients had significantly higher values of hemoglobin (*p*=0.036), cobalamin (*p*=0.023), albumin (*p*=0.005), parathyroid hormone (PTH) (*p*=0.038), and vitamin D (*p*=0.005), and significantly lower values of creatinine (*p*=0.006) and potassium (*p*=0.011), compared to controls (Table 1). Further, gender-matched analyses of TMD patients and controls showed that in the TMD patient group only albumin was significantly higher in both women ((*p*=0.017) and men (*p*=0.026). In women with TMD, significantly higher values of hemoglobin (*p*=0.045), cobalamin (*p*=0.020), PTH (*p*=0.040), and vitamin D (*p*=0.002), as well as significantly lower values of creatinine (*p*=0.018) and potassium (*p*=0.002), were observed. In men with TMD, significantly higher values of TSH (*p*=0.040) were observed (Table 2). Further details of serum levels outside normal reference values in both groups are presented in Table 3. Low levels of vitamin D were significantly correlated (*p*=0.002) with high levels of PTH in the control group, however nonsignificant in the patient group (Figure 2).

### 3.3. Medications

Regular medications used by the patients were paracetamol (*n* = 28), NSAIDs (*n* = 23, hereunder celecoxib in one patient), opioids (*n* = 20, hereunder strong opioids in 5 patients and weak opioids in 17 patients), antidepressants (*n* = 15, hereunder tricyclic antidepressants in 7 patients and selective antidepressants in 10 patients), zopiclone (*n* = 7), clonazepam (*n* = 3), gabapentinoids (*n* = 6, hereunder gabapentin in 4 patients and pregabalin in 2 patients), carbamazepine (*n* = 1), and topiramate (*n* = 1). Serum levels of calcium and creatinine were normal in patients who used celecoxib, pregabalin, and topiramate and were within normal reference values. One patient who used carbamazepine had slightly elevated levels serum TfR (5.7 mg/L) and normal serum levels of serum Hb.

## 4. Discussion

Based on the results of the present study, we were unable to associate any severe systemic disease, malnutrition, or systemic inflammation with TMD. We performed nineteen different, common diagnostic serum analyses determining essential proteins, hormones, ions, and vitamins, and most of the TMD patients and controls showed values within normal biologic range. Medications used by the patients were not found to have any major impact on serum analyses. Findings from our study support results from the OPPERA project, suggesting that TMD is a complex disorder influenced by psychosocial factors and pain sensation rather than being a state of disease indicated by analyses of serum compounds [3]. Supporting observations have also been reported, currently, regarding levels of vitamin D and calcium [6]. Nonsupporting studies have shown a high prevalence of malnutrition including deficiencies in vitamin D, vitamin B, and iron in a population of TMD patients [7], as well as a high prevalence of low serum vitamin B, folate, and iron [8].

An unexpected result revealed that the control group had significantly lower values of vitamin D compared to the TMD group. In both groups, deficiency in D vitamins was commonly observed. A high prevalence of vitamin D deficiencies in both TMD patients and healthy controls [6], as well as healthy individuals [17], has also been reported previously. Nearly one half of TMD patients in Saudi population were also reported to have low vitamin D levels, although there was no control group for comparison [7]. In nonspecific musculoskeletal persistent pain, vitamin D deficiency was observed in the majority of all patients, implying that there is a link between chronic pain and vitamin D deficiency [18]. Prevalence of vitamin D deficiencies has been observed to be higher in patients with chronic pain [11] and has also been associated with chronic widespread pain in a meta-analysis [19]. Vitamin D supplements, in some studies, have been linked with alleviation of chronic pain [11]. Vitamin D has also been suggested to have a function in the maintenance of chronic pain and associated comorbidities through hormonal, immunological, and neurological influences [12]. In a randomized controlled trial of fibromyalgia patients, normalization of vitamin D levels was associated with a decrease in pain intensity measured on a visual analog scale (VAS) [20]. On the other hand, results from a meta-analysis failed to prove the effect of vitamin D supplementation on pain in subjects with chronic musculoskeletal pain [21]. It also appears that vitamin D deficiency is quite common in the healthy population and varies throughout the year [17, 22]. The difference of lower vitamin D levels in our control group could potentially be explained by three factors. First is the geographic factor; all subjects in the control group lived in Bergen, where the number of hours of sun per year is very low, while the TMD patients were from all over the country of Norway. The second factor is that TMD patients probably consult with their GP more often and get their nutritional serum levels analyzed more often compared to the healthy individuals. The third factor is that more subjects in the TMD group probably take overdoses of nutritional supplements, as confirmed by the outcome from our investigation.

Parathyroid hormone (PTH) was significantly higher in the TMD group of the present study. However, both elevated and lowered levels of PTH were observed in the TMD patient group on the individual level. A high level of PTH reflects hyperparathyroidism, while low levels reflect hypoparathyroidism. Elevated levels of PTH in TMD patients compared to healthy controls have previously been reported [6], where the majority of TMD patients had elevated PTH levels, primarily described as a response to low vitamin D levels. The level of PTH in depressed patients has also been demonstrated to be significantly higher compared to controls [23]. On the other hand, a clinical study examining hyperparathyroidism in patients with fibromyalgia, widespread pain, and localized musculoskeletal pain concluded there were no differences in prevalence between the groups nor compared to the general population [24]. A few patients in the present study also had elevated levels of free thyroxine (FT4), which may reflect hyperthyroidism. A recent clinical case-control study reported a significantly higher prevalence of TMD symptoms as well as the severity of symptoms in patients with Hashimoto thyroiditis (HT) compared to healthy control subjects [25]. HT goes through several stages from hyperparathyroidism to hypoparathyroidism, and its main symptoms are like TMD, including musculoskeletal pain and stiffness. The fact that thyroid hormones play an important role in muscle function [26] is supported by the common occurrence of neuromuscular symptoms [27] and musculoskeletal disorders [28] in patients with thyroid dysfunctions. Thyroid disease has also previously been associated with idiopathic tongue pain in a clinical study [29]. Observed levels of PTH in the present study may possibly reflect some prevalence of parathyroid disturbances or disturbances in the hormonal thyroid-regulating pathways in the TMD group. However, there was no overall significant difference in thyroid stimulating hormone (TSH), nor in FT4, between the TMD patient group and the control group. An exception was observed in the subgroup of male TMD patients, who had significantly higher levels of TSH compared to the subgroup of male controls.

Elevated PTH is often seen in association with low vitamin D, leading to elevated calcium absorption from bone [22]. Similarly, we observed a significant negative association between PTH levels and vitamin D levels in the control group but not in the patient group. The observed association may be explained by the fact that vitamin D levels were lower in the control group, resulting in higher stimulation of excretion of PTH, compared to the patient group. A supporting observation from a laboratory database study, examining the association between serum PTH and vitamin D levels in 19,172 subjects in the Israeli population, concluded that vitamin D levels had to be below the reference value of 50 nmol/L to sufficiently elevate PTH levels [30]. A noteworthy finding considering vitamin D and PTH levels in the present study was that most subjects in both groups had normal values of calcium. Elevated vitamin D and PTH in the presence of normal calcium values were also observed in a previous study of TMD patients [6]. A possible explanation could be that normalized levels and intake of calcium may suppress the increase of secretion of PTH, when vitamin D is low [17].

There were no significant differences in CRP levels between the TMD group and the control group. Mean CRP levels have previously been observed within the normal range in patients with TMJD and were not affected by pain [10]. The fact that most TMD patients have normal values of CRP means that the pain intensity is probably not directly associated with inflammation. However, some patients in the present study had elevated CRP levels ranging from 5 to 20 mg/L that may potentially be enough to reflect a local inflammation. In a radiographic CT and MRI study of patients with rheumatoid arthritis (RA), mean CRP levels were between 10 and 20 mg/L, and the levels of CRP were also correlated with inflammation in the TMJ [9]. In other studies of patients diagnosed with RA, higher levels of CRP were correlated with resorbed condyles in the TMJ, seen on CT [31], and low jaw opening capacity [32]. In a large population of adults in the United States, elevated serum CRP was positively associated with pain and headache when assessed by questionnaires [33]. The fact that low income also was associated with pain and not with CRP indicates that CRP levels contributed to increased pain independent of pain-related social factors [33]. Albumin, which functions as a carrier protein and has a role in maintaining colloid osmotic pressure, was significantly higher in the TMD group. Elevated levels of albumin normally reflect dehydration. Moreover, high albumin levels have previously been associated with metabolic syndrome [34]. The fact that high levels of albumin have previously been negatively associated with pain [33] and significantly lower levels of inflammatory cytokines in healthy older subjects [35] indicates that high levels of albumin are not related to pain disorders. Since albumin was high in our group of TMD patients, systemic inflammation was also less likely.

Another indicator of high doses of nutritional supplementation in our group of TMD patients was significantly higher levels of cobalamin, possibly due to vitamin B12 supplementation. On the other hand, 10% of the TMD patients had elevated levels of homocysteine, even though there were no statistical differences in mean levels of homocysteine compared to the control group. High homocysteine levels reflect low levels of vitamin B12, vitamin B6, and folate, and the observed elevated levels in some patients may potentially be explained by the possibility of reduced intake of diet sources in the patient group because of jaw impairment and pain from chewing. However, there was no statistical difference in serum folate levels between the TMD patients and controls. A previous study of serum analyses in TMD patients reported a high prevalence of low vitamin B levels, including folate and vitamin B complex [7]. Similarly, a high prevalence of low vitamin B levels in patients with complex TMJ problems, including vitamin B1, vitamin B6, vitamin B12, and folate, was shown in another study [8].

In the TMD group, we observed some patients with elevated levels of transferrin receptor (TfR) and low erythrocyte volume fraction (EVF), which potentially could indicate iron deficiency or an increase in erythropoiesis [36–38]. Iron deficiencies have previously been observed in TMD patients [7, 8]. Although, mean values of TfR and EVF in the present study did not statistically differ between TMD patients and controls, and the fact that hemoglobin was significantly higher in the TMD patient group supports the explanation of higher erythropoiesis. Serum TfR has previously been observed to be elevated in children with juvenile chronic arthritis, without any correlation to serum transferrin or ferritin levels, indicating that serum TfR is an inadequate indicator of iron levels in the presence of chronic inflammation [36].

The levels of creatinine were significantly lower in the TMD patient group. Creatinine is a degradation product of creatine phosphate, which has an important role in muscle function and fast energy production [39]. Low levels of serum creatinine may reflect muscle atrophy and has also been directly associated to low bone mineral density in subjects with normal renal function [40]. In a randomized controlled trial of patients with fibromyalgia, creatine supplementation resulted in significantly improved function of muscle; however, no improvements in pain or psychosocial factors were observed [41].

Potassium was significantly lower in the TMD group. Potassium homeostasis is regulated by renal and extrarenal mechanisms, affected by acid-base balance and hormonal regulation by epinephrine, insulin, and aldosterone [42]. The uptake and release of extracellular potassium is mainly mediated by the skeletal muscles through several *K*+ channels [43], contributing to maintain the extrarenal homeostasis. The fact that abnormal *K*+ channel activity has a role in chronic pain [44] makes it reasonable to suspect that changes in potassium homeostasis may affect the function of *K*+ channels in painful disorders. Despite the presence of statistically significantly lower levels of potassium in our group of TMD patients, the observed difference probably has no clinical value since most patients and controls had normal potassium levels. A possible explanation of lower potassium levels in the patient group may be due to lower intake of fluid or electrolyte status. However, sodium levels were normal in all patients and did not statistically differ from the control group, indicating normal electrolyte balance.

The prevalence and severity of TMD is higher in women compared to men [1]. Results from a recent meta-analysis showed that women had a two-time higher risk in development of TMD [4]. In a clinical study of TMD patients, women showed significantly higher pain intensity on the VAS compared to men, as a respond to palpation of the masticatory muscles as well as the TMJ [5]. Results from the OPPERA study have shown that pain sensitivity, in the majority of all pain measurements, is significantly higher in women compared to men [45].

Because of the higher prevalence of TMD in women, we found it interesting to reveal gender differences in the serum analyses in both groups. The gender-matched serum analyses showed significantly higher levels of hemoglobin, cobalamin, PTH, and vitamin D, as well as significantly lower levels of creatine and potassium only in women in the TMD group, compared to the control group. Albumin levels were significantly higher in both men and women in the TMD group, and TSH was significantly higher only in men in the TMD patient group, compared to the control group. The results showed that most of the observed differences in serum analyses, for both groups, were in the subgroup of women. However, the observed gender differences may be affected by the fact that men were a very small population sample comprising only 9 subjects in each group. More research must be carried out to fully examine the possibility of an association between gender and serum levels in TMD.

The present study is one of the few studies on serum analyses in TMD patients comparing levels in healthy individuals. To our knowledge, this is the first study to use as many as 19 different variables at the same time. Despite a relatively small study sample, the results of our study may contribute toward assessing and mapping risk factors and characteristics of TMD. One limitation of the study was the fact that the serum samples were taken at all seasons of the year, while levels of some determinants, e.g., vitamin D and PTH, may have some seasonal variability in the Scandinavian countries. The fact that there was some geographical difference between the TMD patient group and the control group may also have had an effect on the results. The possibility of cultural, diet-related, and socioeconomic factors affecting serum levels of the presented determinants in our study, as well as other similar reports, should also be kept in mind. Another limitation is that ultrasound was not used for diagnostics of TMD, which has been tried but had too many obstacles to pass and therefore was not used. The gold standard for diagnostics of TMJ disease is magnetic resonance imaging (MRI) together with a clinical investigation for function and pain. However, ultrasound for diagnostics has recently been shown to be effective in the limb muscle and shoulders [46, 47].

## 5. Conclusion

In the present study, no clear indication of systemic disease or malnutrition in the TMD patient group was seen. The results from the serum analyses were mostly within normal reference values, which resulted in rejection of our hypothesis. All observed deficiencies in both groups were weak. One of the most surprising results was that vitamin D levels were lower in the control group compared to the TMD group. Despite minor observed differences in TMD patients, as compared with healthy subjects, we conclude that serum analyses should be not be used as a biomarker of TMD nor as a diagnostic tool for an individual subject with TMD. As an outcome from our clinical investigation, we observed a small group within the TMD group which likely took high doses of vitamin supplements, which contributed to elevated serum levels in some variables. Due to a relatively low number in the study population, more research is warranted to clarify the relationship between different serum determinants and the etiology and maintenance of TMD.

## Figures and Tables

**Table 1:** Serum analyses resulted in nineteen different determinants common in diagnostics. Second column shows normal values for women and men at all ages. Most TMD patients and controls had values within normal biologic range. TMD patients had significantly higher values of hemoglobin (*p*=0.036), cobalamin (*p*=0.023), albumin (*p*=0.005), PTH (*p*=0.038), and vitamin D (*p*=0.005) and significantly lower values of creatinine (*p*=0.006) and potassium (*p*=0.011) compared to controls.

Serum analyses	Reference values	TMD			Control			*p*-value (paired *t*-test)
		Mean	±SD	Range	Mean	±SD	Range	(^*∗*^ = sign.)
Hemoglobin	*W*: 11.7–15.3, *M*: 13.4–17.0 g/dL	14.1	1.28	8.1, 16.2	13.8	0.83	11.5, 16	0.036^*∗*^
EVF	*W*: 0.35–0.46, *M*: 0.40–0.50	0.41	0.04	0.28, 0.48	0.41	0.02	0.35, 0.48	0.325
MCV	82–98	91.2	4.66	76, 102	90.1	3.62	83, 100	0.299
Homocysteine	<15 umol/L	11.1	3.79	5.8, 28.0	11.3	3.03	5.7, 24.2	0.459
Transferrin R	*W*: 1.9–4.4, *M*: 2.2–5.0 mg/L	3.27	1.78	1.7, 14.6	2.98	0.80	1.7, 5.9	0.103
TSH	0.4–4.5 mlE/L	1.63	0.83	0.01, 4.31	1.80	0.98	0.14, 4.72	0.191
FT4	9.5–22.0 pmol/L	16.0	3.13	7.8, 29	15.7	2.26	11.5, 27.0	0.266
Cobalamin	175–700 pmol/L	422	194.8	167, 1322	363	124.9	129, 702	0.023^*∗*^
Folate	>8 nmol/L	20.7	10.41	7.7, 45.3	20.2	7.38	7.5, 45.3	0.450
CRP^*∗*^ (<1 = 1)	<5 mg/L	2.43	3.09	1.0, 14.0	2.10	2.84	1.0, 20.0	0.268
Creatinine	*W*: 45–90, *M*: 60–105 umol/L	65.6	10.47	45, 93	69.7	12.14	49, 108	0.006^*∗*^
Estimated GFR	>90 mL/min/l	>60	—	—	>60	—	—	—
Sodium	137–145 mmol/L	140.1	1.48	137, 144	140.0	1.39	136, 144	0.227
Potassium	3.5–5.0 mmol/L	4.0	0.25	3.2, 4.6	4.1	0.31	3.4, 5.3	0.011^*∗*^
Calcium	2.20–2.55 mmol/L	2.40	0.09	2.21, 2.63	2.40	0.08	2.15, 2.59	0.456
GT	*W*: <40y = 10–45, >40y = 10–75 U/L *M*:<40y = 10–80, >40y = 15–115 U/L	22.2	19.63	8.0, 145.0	17.9	12.20	6.0, 72.0	0.078
Albumin	<39y: 39–50, 40–69y: 39–48, >70y: 36–48 g/L	46.3	2.94	40.0, 53.0	45.1	1.96	40.0, 49.0	0.005^*∗*^
PTH	1.3–6.8 pmol/L	3.4	1.53	0.9, 8.2	2.9	1.09	1.0, 6.0	0.038^*∗*^
Vitamin D	50–113 nmol/L	72.4	26.93	19.0, 187.0	61.1	18.68	22.0, 127.0	0.005^*∗*^

**Table 2:** Gender-matched serum analyses: Results from serum analyses showed mean levels statistically differed between TMD patients and controls, divided into subgroups of women and men. The second column shows normal values for women and men at all ages. Significantly higher levels of hemoglobin (*p*=0.045), cobalamin (*p*=0.020), albumin (*p*=0.017), PTH (*p*=0.040), and vitamin D (*p*=0.002) were observed in women in the TMD group. Significantly lower levels of creatinine (*p*=0.018) and potassium (0.002) were observed in women in the TMD group. Significantly higher levels of TSH (*p*=0.040) and albumin (*p*=0.026) were observed in men in the TMD group. The number of men was considerably smaller in both groups.

Gender-matched serum analyses	Reference values	TMD (*n* = 60)			Control (*n* = 60)			*p*-value (paired *t*-test)
		Mean	±SD	Range	Mean	±SD	Range	(^*∗*^ = sign.)
*Hemoglobin*								
Women (*n* = 51 + 51)	11.7–15.3 g/dL	13.9	1.22	8.1, 15.7	13.6	0.70	11.5, 14.7	0.045^*∗*^
Men (*n* = 9 + 9)	13.4–17.0 g/dL	15.2	1.13	13.4, 16.2	14.9	0.62	14.0, 16.0	0.288

*TSH*								
Women (*n* = 51 + 51)	0.4–4.5 mlE/L	1.52	0.82	0.01, 4.31	1.80	1.04	0.14, 4.72	0.111
Men (*n* = 9 + 9)	0.4–4.5 mlE/L	2.24	0.59	1.25, 3.34	1.84	0.52	1.17, 2.49	0.040^*∗*^

*Cobalamin*								
Women (*n* = 51 + 51)	175–700 pmol/L	425	210.0	167, 1322	355	128.4	129, 702	0.020^*∗*^
Men (*n* = 9 + 9)	175–700 pmol/L	401	60.67	310, 455	403	100.3	291, 568	0.483

*Creatinine*								
Women (*n* = 51 + 51)	45–90 umol/L	62.8	8.01	45, 81	66.2	8.77	49, 84	0.018^*∗*^
Men (*n* = 9 + 9)	60–105 umol/L	81.3	8.93	68, 93	88.9	9.97	74, 108	0.098

*Potassium*								
Women (*n* = 51 + 51)	3.5–5.0 mmol/L	4.0	0.25	3.2, 4.6	4.1	0.30	3.4, 5.3	0.002^*∗*^
Men (*n* = 9 + 9)	3.5–5.0 mmol/L	4.1	0.21	3.9, 4.5	4.0	0.33	3.5, 4.5	0.267

*Albumin*								
Women (*n* = 51 + 51)	Age dependent <39y: 39–50, 40–69y: 39–48,	45.9	2.98	40.0, 53.0	44.9	1.91	40.0, 49.0	0.017^*∗*^
Men (*n* = 9 + 9)	>70y: 36–48 g/L	48.0	2.08	45.0, 51.0	46.6	1.59	44.0, 48.0	0.026^*∗*^

*PTH*								
Women (*n* = 51 + 51)	1.3–6.8 pmol/L	3.5	1.58	0.9, 8.2	2.9	1.03	1.0, 5.2	0.040^*∗*^
Men (*n* = 9 + 9)	1.3–6.8 pmol/L	2.7	1.10	1.1, 4.2	2.58	1.39	1.4, 6.0	0.385

*Vitamin D*								
Women (*n* = 51 + 51)	50–113 nmol/L	75.3	26.23	19.0, 187.0	61.8	19.78	22.0, 127.0	0.002^*∗*^
Men (*n* = 9 + 9)	50–113 nmol/L	55.8	26.21	21.0, 111.0	57.6	11.02	42.0, 72.0	0.426

**Table 3:** Elevated and lowered values: observed number and percent of patients and controls with elevated and lowered values of the different determinants from the serum analyses. Mild to moderate vitamin D level deficiency was seen in 11 TMD patients compared to 18 controls. Furthermore, we observed marginally elevated levels of CRP (*n* = 7) and elevated levels of transferrin receptor (*n* = 7) and homocysteine (*n* = 6), as well as lowered levels of erythrocyte volume fraction (EVF) (*n* = 5), in the TMD group. FT4 was elevated in 3 patients and lowered in one patient, while parathyroid hormone (PTH) was elevated in 2 patients and lowered in 4 patients.

Elevated and lowered values	TMD				Control			
	Elevated		Lowered		Elevated		Lowered	
	*n*	%	*n*	%	*n*	%	*n*	%
Hemoglobin	1	1.7	2	3.3	0	0.0	1	1.7
EVF	1	1.7	5	8.3	0	0.0	0	0.0
MCV	1	1.7	3	5.0	4	6.7	0	0.0
Homocysteine	6	10.0	0	0.0	3	5.0	0	0.0
Transferrin R	7	11.7	3	5.0	3	5.0	2	3.3
TSH	0	0.0	2	3.3	1	1.7	1	1.7
FT4	3	5.0	1	1.7	1	1.7	0	0.0
Cobalamin	5	8.3	1	1.7	1	1.7	1	1.7
Folate	0	0.0	2	3.3	0	0.0	1	1.7
CRP	7	11.7	0	0.0	4	6.7	0	0.0
Creatinine	0	0.0	0	0.0	1	1.7	0	0.0
Estimated GFR	0	0.0	0	0.0	0	0.0	0	0.0
Sodium	0	0.0	0	0.0	0	0.0	1	1.7
Potassium	0	0.0	1	1.7	1	1.7	1	1.7
Calcium	1	1.7	0	0.0	3	5.0	1	1.7
GT	2	3.3	5	8.3	0	0.0	7	11.7
Albumin	9	15.0	0	0.0	0	0.0	0	0.0
PTH	2	3.3	4	6.7	0	0.0	1	1.7
Vitamin D	3	5.0	11	18.3	1	1.7	18	30.0

## Data Availability

Data used to support the findings of this study are available from the corresponding author upon request.
